# University Students Purchasing Food on Campus More Frequently Consume More Energy-Dense, Nutrient-Poor Foods: A Cross-Sectional Survey

**DOI:** 10.3390/nu13041053

**Published:** 2021-03-24

**Authors:** Megan C Whatnall, Zhao Min Soo, Amanda J Patterson, Melinda J Hutchesson

**Affiliations:** 1 School of Health Sciences, College of Health, Medicine and Wellbeing, University of Newcastle, Callaghan, NSW 2308, Australia; megan.whatnall@newcastle.edu.au (M.C.W.); zhao.soo@uon.edu.au (Z.M.S.); Amanda.patterson@newcastle.edu.au (A.J.P.); 2 Priority Research Centre for Physical Activity and Nutrition, University of Newcastle, Callaghan, NSW 2308, Australia; 3 Hunter Medical Research Institute, New Lambton Heights, NSW 2305, Australia

**Keywords:** university, college, dietary intake, diet quality, purchasing behaviour

## Abstract

University food environments are typically dominated by unhealthy food choices. The aim was to investigate associations between on-campus food purchasing behaviours and dietary intake in an Australian university student sample. An online cross-sectional survey was conducted in 2017–2018 with students (*n* = 362, 71.0% female, mean age 27.5 ± 10.5 years) from the University of Newcastle, Australia. On-campus food purchasing behaviours (purchasing frequency and weekly expenditure), dietary intake (diet quality and percentage energy/day from energy-dense, nutrient-poor (EDNP) foods) and sociodemographic and student characteristics (e.g., time spent on campus) were measured. Linear regression was used to explore associations between food purchasing behaviours and dietary intake, adjusted for potential confounders. Mean percentage energy/day from EDNP foods was 31.7 ± 14.4. Mean diet quality score was 32.6 ± 10.2 out of 73. Higher percentage energy/day from EDNP foods was associated with higher weekly expenditure (β = 0.203, *p* < 0.001) and more frequent purchase (β = 18.041, *p* < 0.001 for ≥4 times a week vs. never) of food/drinks on campus. Diet quality was not significantly associated with purchase frequency or expenditure (*p* > 0.05). Findings are supportive of changes being made to university food environments, as a strategy to improve dietary intake among university students.

## 1. Introduction

University food environments are typically dominated by unhealthy food choices with very limited healthy choices. For example, an observational study of 57 food outlets across six campuses of a metropolitan university in New Zealand categorized only 11% of outlets as healthy, based on the proportion of healthy and unhealthy food and drink products available [1]. In the USA, an audit of 263 dining outlets across 15 tertiary education institutions reported that only 40% offered healthy main dishes [2]. Further, a high proportion of university students consume excess energy-dense, nutrient-poor (EDNP) foods, and below recommendations for nutrient-rich foods such as whole-grain foods and vegetables. For example, cross-sectional studies from the USA, continental Europe and the UK show around 22–37% of students regularly consume fast foods or confectionary (between four days per week and several times per day) [3,4,5]. Studies of Australian and UK university students have also reported that 86–92% of students consume below the recommendations for daily fruit and vegetables serves [3,6]. This is problematic as a large proportion of the population globally attend university, and the university environment should be health-promoting [7] rather than potentially contributing to poor dietary intakes and associated poor health and other outcomes. 

There are many factors which influence dietary intake among university students. Some of the key barriers to eating well that have been identified include limited time to plan and prepare foods, limited budget available to spend on food, and social and environmental influences such as unhealthy dietary intakes of peers and minimal kitchen facilities in common living arrangements such as share housing and on-campus accommodation [8,9,10]. Additionally, university students are often juggling their studies alongside other work, family, sport, and social commitments, and many are in the young adult life stage, which is a developmental stage for health behaviours [8,10]. These are all factors which increase university students’ vulnerability to having poor dietary intake. Due to many of these factors, university students may also be more influenced by unhealthy food environments, such as the typical university food environment.

However, there has been minimal research exploring the associations between purchasing food and drinks on campus and students’ dietary intake. Pelletier et al., in a cross-sectional study among approximately 1000 USA university students, found that students who purchased food and beverages on campus more frequently had higher intakes of fat and added sugars and less frequent breakfast consumption [11]. Additionally, Roy et al. found that among a sample of 103 university students of a large metropolitan Australian university, students’ diet quality scores were significantly lower (i.e., less healthy) relative to consuming food and/or beverages on campus more often [12]. While the available evidence suggests that there is a link between purchasing food and drinks on campus and dietary intake among university students, there is a limited number of studies available and, in particular, a limited number of recent studies or studies which have assessed expenditure in relation to dietary intake. Further, university food environments and students purchasing habits likely differ between countries due to differences in living arrangements, food provision, and financial situation. Differences are also likely to exist between universities that are situated in metropolitan compared with urban and regional locations, due to factors such as proximity to external food and beverage outlets. Additionally, university environments and food purchasing and consumption behaviours are changing over time, and therefore, research of this nature should be conducted regularly to capture these changes. As such, there is a need for further studies to explore associations between the university food environment and dietary intake among university students in different settings. 

The aim of this study was to explore the associations between on-campus food purchasing behaviours (frequency of purchase, weekly expenditure) and dietary intake among a sample of university students at a large urban Australian university.

## 2. Materials and Methods

### 2.1. Study Design

This study was an online cross-sectional survey conducted at the University of Newcastle (UON), Australia. The survey was hosted in Qualtrics (https://www.qualtrics.com/ accessed on 1 August 2017) and was run between 17 October 2017 and 12 March 2018. The survey’s primary aim was to determine students’ dietary intake and opinions about the cost and availability of foods and beverages at University of Newcastle campuses. The survey included 158 questions in total, including the following sections: demographic characteristics (22 questions), questions on purchasing behaviour and opinions relative to the on-campus food environment (10 questions), dietary intake (120 questions), and food security (6 questions). A sub-set of these questions were included in the current study. The study conduct and reporting is compliant with STROBE-nut guidelines [13]. Approval for this study was obtained from the UON Human Research Ethics Committee (H-2017-0323). All participants gave informed consent prior to participating. 

### 2.2. Participants and Recruitment

Students enrolled at the UON, a large urban university in New South Wales (NSW), Australia were eligible to participate. Participants were recruited from two campuses, including the main campus in Newcastle and the Ourimbah campus. Students who were enrolled in courses at either campus during semester two of 2017 were eligible (~28,000 students). Convenience sampling was used for recruitment. Recruitment was completed in two stages; firstly, students who had completed a different online survey in 2017 (*n* = 2803) and consented to being re-contacted for future research (*n* = 1582) were sent an initial email invitation and reminder one week later. The study was also advertised via the University’s social media pages (e.g., Facebook), and digital signage displayed on campus (e.g., computer screen savers) for one month, and members of a consultative group of staff and students who guide health promotion initiatives (University Health Promotion Working Group) were contacted two times via email with the request to distribute study details amongst students. Participants could enter a prize draw on survey completion, including the chance to win one of ten gift vouchers valued at AUD 100. Informed consent was obtained from all participants prior to their participation in the survey.

### 2.3. Measures

#### 2.3.1. On-Campus Food Purchasing Behaviours (Independent Variables)

On-campus food purchasing behaviour was assessed using five questions developed for this study based on a previously conducted survey at an Australian university [14]. Two questions were included in the present analysis, including how often participants purchase food or beverages on campus (categorical question, 6 response options ranging from never to ≥4 times a week) and how much money participants normally spend on food and beverages purchased on campus each week (continuous question, reported to the nearest dollar between AUD $0 to $300). The survey also included five questions on participants’ opinions and satisfaction with the on-campus food environment; however, these are outside the scope of the current analysis.

#### 2.3.2. Dietary Intake (Dependent Variables)

The validated Australian Eating Survey Food Frequency Questionnaire (AES FFQ) [15,16] was used to assess dietary intake. The AES FFQ is a 120-item semi-quantitative FFQ assessing usual intake over the preceding 3–6 months. The dietary intake measures used in this analysis included diet quality and percentage energy per day from energy-dense, nutrient-poor (EDNP) foods. Diet quality was derived from the AES FFQ responses using the Australian Recommended Food Score (ARFS). The ARFS is calculated from a subset of 70 items from the AES FFQ relating to intake of each of the eight subscales, including vegetables, fruit, dairy foods, breads and cereals, meat and flesh foods, non-meat and flesh protein foods, spreads and sauces, and water. The ARFS is calculated by summing the points for each item, with most items assigned one point for a consumption frequency of once per week or more, or zero for consumption of less than once per week. Total ARFS ranges from 0 to 73 points, with a higher score indicating higher diet quality, including greater variety, more optimal nutrient intakes, and dietary intake more closely in line with the Australian Dietary Guidelines. The percentage of energy per day from EDNP foods was calculated as the sum of percentage energy per day from a subset of 50 items from the AES FFQ from nine sub-groups, including packaged snacks (e.g., potato chips), sweetened drinks, confectionary, baked sweet products (e.g., sweet pastries), fatty meats (e.g., sausages), fried/takeaway, spreads and sauces, alcoholic drinks, and miscellaneous (soups, coffee, and tea). 

#### 2.3.3. Sociodemographic and Student Characteristics 

Data were captured on age, gender, country of birth, marital status, Aboriginal or Torres Strait Islander (ATSI) descent, living situation, hours of paid work, and sources of financial support (parents/guardians, partner, government, scholarship, other or none) to describe sociodemographic characteristics of participants. Type of degree (enabling course, undergraduate, postgraduate (research or coursework), or English language course for international students), number of years studying, faculty of study, whether they were a domestic or international student, and how many hours they spend on campus each week (1–10, 11–20, 21–30, 31–40 or more than 40 h) were captured to describe student-specific characteristics of the sample.

### 2.4. Statistical Analysis

Stata statistical software (version 14.2) was used for all analyses. Sociodemographics, student characteristics, on-campus food purchasing behaviours, and dietary intake data are reported as mean and standard deviation (SD) or median and interquartile range (IQR) for continuous data and percentages for categorical data. A total of 513 individuals consented to complete the survey, with 437 meeting eligibility criteria. A total of 362 students were included in the analysis, with participants excluded if they had missing/incomplete data for on-campus food purchasing (*n* = 28) or dietary intake (*n* = 42). A further five participant responses for on-campus food purchasing were considered implausible and were excluded, that is, they indicated that they purchase food or beverages on campus but spend zero dollars per week on purchases made. Unadjusted linear regression models were used to explore the associations between on-campus food purchasing behaviour (frequency and expenditure of purchase) and dietary intake (ARFS and percentage energy per day from EDNP foods), and the associations of these with sociodemographic and student characteristics to identify potential confounders. Adjusted linear regression models were then used to explore the associations between on-campus food purchasing behaviour and dietary intake, adjusted for the individual sociodemographic and student characteristics that were statistically significant in the unadjusted models to account for potential confounding. Statistical significance for the identification of potential confounders was set at *p* < 0.2, and *p* < 0.05 for the adjusted models.

## 3. Results

### 3.1. Summary of Sample Characteristics

The mean (SD) age of participants was 27.5 (10.5) years, and most were female (71.0%) (Table 1). Three quarters of the sample were undergraduate students (74.3%) and reported that they receive financial support (e.g., from parents/guardians or government support) (72.1%). Students from all faculties across the university participated and were varied in the number of years they had been studying. Students predominantly lived in rented accommodation (43.9%) or their parents’ home (34.8%). Sixty-two percent reported that they spend up to 20 h per week on campus. 

### 3.2. Summary of On-Campus Food Purchasing Behaviours and Dietary Intake

The majority of students reported that they purchase food and/or beverages on campus once a week or more often (58.8%) (Table 2). Students’ median (IQR) expenditure on food and/or beverage purchases on campus was AUD 17.50 (10–25) per week. The mean (SD) ARFS was 32.6 (10.2) and the mean (SD) percentage of energy/day from EDNP foods was 31.7 (14.4). 

### 3.3. Associations between On-Campus Food Purchasing Behaviours with Dietary Intake

Results of the unadjusted and adjusted linear regression models exploring on-campus food purchasing behaviours with ARFS and percentage energy from EDNP foods are presented in Table 3 and Table 4, respectively. In the unadjusted models, lower ARFS was associated with more frequent purchasing of food and beverages on campus (*p* = 0.047); however, in the adjusted models, ARFS was not significantly associated with frequency of or expenditure on purchasing food and beverages on campus (*p* > 0.05). In the unadjusted models for EDNP foods, higher percentage of energy from EDNP foods was associated with more frequent purchasing of food and beverages on campus and greater weekly expenditure on food and beverages on campus (*p* < 0.001). These associations remained significant in adjusted models, controlled for sociodemographic and student characteristics (*p* < 0.001). 

## 4. Discussion

This study presents new findings on the associations between on-campus food purchasing behaviours (frequency and expenditure) and dietary intake among an Australian sample of university students. Greater frequency and expenditure of purchasing food and beverages on campus were found to be associated with a higher intake of energy-dense, nutrient-poor foods but were not significantly associated with diet quality score. These findings suggest that the university food environment may influence students’ intake of EDNP foods, and therefore, more attention is needed to improve the healthiness of university food environments. 

The current study found that the proportion of students’ energy intake from EDNP foods was significantly higher with more frequent purchasing of food and beverages on campus, with a difference of +18.0% of energy from EDNP foods in those purchasing food and beverages on campus four or more times per week compared with never. The association with diet quality assessed using the ARFS, however, was not found to be significant. These findings show some consistency with the available evidence, particularly the study by Pelletier et al. which found that among 1059 USA university students, those who purchased food on campus three or more times per week compared with less than three times were consuming +1.5% of energy from fat and +3.8 teaspoons of added sugars per day [11]. Further, Roy et al. found that among Australian university students (*n* = 103) who provided dietary intake data via 5-day weighed food records, those who purchased six or more foods on campus over the five days compared with two or less had a 14.2/100 lower (i.e., less healthy) diet quality score [12]. Although in the current study, no significant difference was identified between frequency of purchasing food and beverages on campus and the ARFS diet quality score, the direction of the association was negative (i.e., lower diet quality with more frequent purchase). It is also important to note that the ARFS is reflective of nutrient-rich core foods only, where the study by Roy et al. used the Healthy Eating Index for Australians (HEIFA-2013) which includes both core and energy-dense, nutrient-poor foods. It could be that some of the differences across studies are due to differences in dietary assessment tools, as well as varying sample sizes. It could also be that frequency of purchasing foods on campus has minimal impact on intakes of nutrient-rich core foods, while significantly increasing intakes of EDNP foods. It was also found in the current study that the differences in EDNP food consumption were only significant for students who purchase foods on campus fortnightly or more often. The dietary intakes of students consuming foods on campus less often would be determined by the off-campus food environment such as home, eating out, and takeaway [17]. Overall, the collective evidence from studies among university students in Western countries demonstrates that frequent consumption of food and beverages from university campuses is associated with poorer dietary intake. 

The proportion of students’ energy intake from EDNP foods was significantly higher as students reported spending more money per week purchasing food and beverages on campus, although the magnitude of the association was much smaller than for frequency of purchase, with a difference of +2% of energy from EDNP foods for every $10 spent. Weekly expenditure was not significantly associated with ARFS. These findings are inconsistent with the general line of thinking that healthier foods are more expensive, and particularly when choosing healthier foods from food outlets. However, the current findings are relative to the foods that are available. That is, across the two university campuses in this study, many of the foods and beverages available are EDNP [18]. The limited availability of healthier foods and therefore limited number of students purchasing these foods may be contributing to the lack of significant findings between ARFS and on-campus food purchasing behaviours in this study. In comparison, Sprake et al. conducted a cross-sectional survey of dietary patterns among 1448 university students from five universities in the UK and found that weekly expenditure on food was significantly associated with two of the four dietary patterns identified [19]. Students in the health-conscious dietary pattern reported spending the most amount of money on food per week, while students in the convenience, red meat, and alcohol dietary pattern reported spending the least. However, this study assessed total food expenditure and was not specific to purchases made on university campuses. Expenditure is an important consideration in this group, as cost of foods is a key determinant of food choices among university students [1,9] and because of the known linkages between cost of foods and socioeconomic status with diet quality [20]. 

The main strengths of this study include the inclusion of a validated dietary assessment tool and consideration of both nutrient-rich core foods and energy-dense, nutrient-poor food intake, as well as exploring two indicators of food purchasing behaviour. Further, a moderate sample size was achieved, and statistical analyses were controlled for potential confounding variables including sociodemographics and student characteristics which may influence on-campus purchasing behaviour and dietary intakes. Although a range of characteristics were considered, such as financial support and living situation, others that were not assessed may also influence purchasing behaviour and dietary intake, such as knowledge and social influences. In terms of limitations, the cross-sectional design means that findings are associative only, and causal relationships between food purchasing behaviours and dietary intake cannot be determined. The sample in this study was a convenience sample and not powered to detect differences in dietary intake based on food purchasing behaviours. Further, the generalisability of findings to other universities depends on the comparability of their on-campus food environments and characteristics of their student populations. The convenience sample included higher proportions of female students, undergraduate students, and students of Aboriginal and Torres Strait Islander descent than the average across Australian universities [21], and higher proportions of female and undergraduate students, and students studying health and medicine and education and arts compared with the overall University of Newcastle student population. The use of convenience sampling and a monetary incentive for participation may have introduced selection bias, and this as well as the sample representativeness should be considered when interpreting the study findings. Additionally, food purchasing behaviours outside the university setting were not assessed in this study, which may have provided greater context to the findings for on-campus food purchasing behaviours and the association with overall dietary intake.

Overall, the findings of this study suggest that healthier university food environments may help university students to achieve healthier dietary intakes. Further research is needed in this space which explores additional factors in university students’ food purchasing behaviour, such as the amount of money available to spend on food, other sources of food procurement, nutrition knowledge and beliefs, and social influences. This further research will extend the understanding of university students’ food purchasing behaviour and dietary intake, and therefore the ability to implement appropriate strategies to create healthier food environments on university campuses and support students to eat well. The findings of this study can be used to inform future research, as well as to advocate for change in creating healthier university food environments. Any future strategies to create healthier university environments would be most effective if implemented within the context of university-wide health promotion initiatives that seek to support students’ health and wellbeing more broadly, as suggested in the Okanagan international charter for health-promoting universities and colleges [7]. For example, implementation of multiple coordinated strategies to ensure greater provision of healthy foods from university food outlets, supporting a culture that encourages healthy lifestyle choices, and individual level strategies that support knowledge and behaviour change.

## 5. Conclusions

This survey identified that university students who more frequently purchase and have greater expenditure on food and beverages on university campus had a higher intake of energy-dense, nutrient-poor foods. Therefore, the university food environment may influence students’ dietary intake negatively, and greater attention to creating a healthy university food environment is warranted. These findings are important as there is little research of this nature. Further studies are needed to explore the associations between student’s food purchasing behaviours and dietary intake in more depth, including influencing factors.

## Figures and Tables

**Table 1:** Demographic characteristics of an Australian university student sample (*n* = 362).

Variable	N or Mean	% or SD
**Gender**		
Male	99	27.4
Female	257	71.0
Another gender identity	6	1.7
**Age (years) (Mean ± SD)**	27.5	10.5
≤20 years old	90	24.9
21–24 years	109	30.1
25–29 years	63	17.4
30–39 years	56	15.5
≥40 years	44	12.2
**Country of birth**		
Australia	310	85.6
Other	52	14.4
**Aboriginal or Torres Strait Islander background**		
Yes	9	2.5
No	353	97.5
**Marital Status**		
Never married	258	71.3
Married	55	15.2
De facto	32	8.8
Separated	4	1.1
Divorced	13	3.6
**Living Situation**		
Renting	159	43.9
Parents home	126	34.8
Own home	44	12.2
On campus	25	6.9
Boarding/Homestay	4	1.1
Irregular	4	1.1
**Receiving financial support**		
Yes	261	72.1
No	101	27.9
**Type of degree**		
Undergraduate	269	74.3
Postgraduate	64	17.7
Enabling Course ^a^	29	8.0
**Faculty of Study**		
Education and Arts	108	29.8
Health and Medicine	100	27.6
Engineering	60	16.6
Science	56	15.5
Business and Law	14	3.9
English Language and Foundation Studies	24	6.6
**Number of years spent studying**		
1st year	134	37.0
2nd year	56	15.5
3rd year	87	24.0
4th year	53	14.6
5th year or later	32	8.8
**Hours spent on campus per week**		
1–10 h	111	30.7
11–20 h	113	31.2
21–30 h	66	18.2
31–40 h	44	12.2
More than 40 h	28	7.7

^a^ Enabling courses are transition to university courses for students not meeting direct entry admission criterion.

**Table 2:** On-campus food purchasing behaviour and dietary intake of an Australian university student sample (*n* = 362).

Variable	Value
**Frequency of purchasing food and beverages on campus, N (%)**	
Never	20 (5.5)
Monthly or less	76 (21.0)
Fortnightly	53 (14.6)
Once a week	91 (25.1)
2–3 times a week	89 (24.6)
≥4 times a week	33 (9.1)
**Weekly expenditure on food and beverages on campus ($AUD), median, IQR ^a^**	17.5, 10–25
**Australian Recommended Food Score (/73), mean, SD**	32.6, 10.2
**Percentage energy from energy-dense, nutrient-poor foods, mean, SD**	31.7, 14.4

^a^ N = 342, i.e., excludes those participants who report that they never purchase food and beverages on campus.

**Table 3:** Linear regression results of on-campus food purchasing behaviour with diet quality in an Australian university student sample (*n* = 362).

	Australian Recommended Food Score (ARFS)					
	Unadjusted Model			Adjusted Model		
	β-Coefficient ^a^	SE	*p*	β-Coefficient ^a^	SE	*p*
**Frequency of purchasing food and beverages on campus**			**0.047**			0.142 ^b^
Reference category = Never						
Monthly or less	0.205	2.529	0.935	0.148	2.498	0.953
Fortnightly	−0.419	2.641	0.874	−0.213	2.620	0.935
Once a week	3.051	2.485	0.220	3.258	2.469	0.188
2–3 times a week	−1.422	2.490	0.568	−0.656	2.496	0.793
≥4 times a week	−1.976	2.852	0.489	−0.599	2.950	0.839
**Weekly expenditure on food and beverages on campus** ^c^	0.001	0.030	0.807	0.029	0.032	0.361 ^d^

^a^ β-Coefficient indicates the increase in the Australian Recommended Food Score (ARFS) per unit increase in the independent variable. ^b^ Model adjusted for age, gender, financial support, type of degree, living situation, international/domestic enrolment, faculty of study, and time on campus. ^c^ Models include *n* = 342, i.e., exclude those participants who reported that they never purchase food and beverages on campus/zero weekly expenditure. ^d^ Model adjusted for age, gender, financial support, Aboriginal or Torres Strait Islander (ATSI) background, living situation, international/domestic enrolment, faculty of study, and time on campus. Significant *p*-values in bold.

**Table 4:** Linear regression results of on-campus food purchasing behaviour with energy-dense, nutrient-poor food intake in an Australian university student sample (*n* = 362)**.**

	Percentage Energy from Energy-Dense, Nutrient-Poor Foods					
	Unadjusted Model			Adjusted Model		
	β-Coefficient ^a^	SE	*p*	β-Coefficient ^a^	SE	*p*
**Frequency of purchasing food and beverages on campus**			**<0.001**			**<0.001 ^b^**
Reference category = Never						
Monthly or less	3.832	3.417	0.263	3.036	3.420	0.375
Fortnightly	7.978	3.569	**0.026**	7.789	3.585	**0.031**
Once a week	8.010	3.358	**0.018**	7.835	3.372	**0.021**
2–3 times a week	14.203	3.365	**<0.001**	14.180	3.412	**<0.001**
≥4 times a week	18.268	3.854	**<0.001**	18.041	4.032	**<0.001**
**Weekly expenditure on food and beverages on campus** ^c^	0.199	0.040	**<0.001**	0.203	0.043	**<0.001 ^d^**

^a^ β-Coefficient indicates the increase in the percentage energy from energy-dense, nutrient-poor foods per unit increase in the independent variable. ^b^ Model adjusted for age, gender, financial support, type of degree, living situation, faculty of study, and time on campus. ^c^ Models include *n* = 342, i.e., exclude those participants who reported that they never purchase food and beverages on campus/zero weekly expenditure. ^d^ Model adjusted for age, gender, financial support, ATSI background, living situation, international/domestic enrolment, and time on campus. Significant *p*-values in bold.
